# Morphotype-Dependent Flow Characteristics in Bicuspid Aortic Valve Ascending Aortas: A Benchtop Particle Image Velocimetry Study

**DOI:** 10.3389/fphys.2017.00044

**Published:** 2017-02-01

**Authors:** Andrew McNally, Ashish Madan, Philippe Sucosky

**Affiliations:** ^1^Department of Aerospace and Mechanical Engineering, University of Notre DameNotre Dame, IN, USA; ^2^Department of Mechanical and Materials Engineering, Wright State UniversityDayton, OH, USA

**Keywords:** hemodynamics, bicuspid aortic valve, aorta, aortopathy, particle image velocimetry

## Abstract

The bicuspid aortic valve (BAV) is a major risk factor for secondary aortopathy such as aortic dilation. The heterogeneous BAV morphotypes [left-right-coronary cusp fusion (LR), right-non-coronary cusp fusion (RN), and left-non-coronary cusp fusion (LN)] are associated with different dilation patterns, suggesting a role for hemodynamics in BAV aortopathogenesis. However, assessment of this theory is still hampered by the limited knowledge of the hemodynamic abnormalities generated by the distinct BAV morphotypes. The objective of this study was to compare experimentally the hemodynamics of a normal (i.e., non-dilated) ascending aorta (AA) subjected to tricuspid aortic valve (TAV), LR-BAV, RN-BAV, and NL-BAV flow. Tissue BAVs reconstructed from porcine TAVs were subjected to physiologic pulsatile flow conditions in a left-heart simulator featuring a realistic aortic root and compliant aorta. Phase-locked particle image velocimetry experiments were carried out to characterize the flow in the aortic root and in the tubular AA in terms of jet skewness and displacement, as well as mean velocity, viscous shear stress and Reynolds shear stress fields. While all three BAVs generated skewed and asymmetrical orifice jets (up to 1.7- and 4.0-fold increase in flow angle and displacement, respectively, relative to the TAV at the sinotubular junction), the RN-BAV jet was out of the plane of observation. The LR- and NL-BAV exhibited a 71% increase in peak-systolic orifice jet velocity relative to the TAV, suggesting an inherent degree of stenosis in BAVs. While these two BAV morphotypes subjected the convexity of the aortic wall to viscous shear stress overloads (1.7-fold increase in maximum peak-systolic viscous shear stress relative to the TAV-AA), the affected sites were morphotype-dependent (LR-BAV: proximal AA, NL-BAV: distal AA). Lastly, the LR- and NL-BAV generated high degrees of turbulence in the AA (up to 2.3-fold increase in peak-systolic Reynolds shear stress relative to the TAV) that were sustained from peak systole throughout the deceleration phase. This *in vitro* study reveals substantial flow abnormalities (increased jet skewness, asymmetry, jet velocity, turbulence, and shear stress overloads) in non-dilated BAV aortas, which differ from those observed in dilated aortas but still coincide with aortic wall regions prone to dilation.

## Introduction

With an incidence rate between 0.5 and 2.0%, the bicuspid aortic valve (BAV) is the most common congenital heart defect and is characterized by the presence of two functional leaflets instead of three in the normal tricuspid aortic valve (TAV) (Roberts, 1970; Ward, 2000). The most common type-I BAV phenotype features two unequally sized cusps and a raphe along the site of fusion on the larger cusp but covers three distinct anatomies, each associated with a different raphe location. While the most prevalent left-right (LR) type-I BAV subtype results from the fusion between the left- and right-coronary leaflets, fusion can also occur between the non- and left-coronary leaflets (NL subtype), or between the right- and non-coronary leaflets (RN subtype) (Sievers and Schmidtke, 2007).

The BAV is a major risk factor for secondary valvular and vascular disease such as calcific aortic valve disease and aortic dilation. Although the susceptibility of BAV patients to such disorders has been described historically as genetic, there is increasing support for a hemodynamic pathway (Barker and Markl, 2011; Girdauskas et al., 2011; Sucosky and Rajamannan, 2013; Atkins and Sucosky, 2014; Sucosky, 2014; Della Corte, 2015). The demonstration of the skewness of the BAV orifice jet (Robicsek et al., 2004; Della Corte et al., 2011) and of its impingement on the anterolateral aortic wall (Robicsek et al., 2004; Hope et al., 2008, 2010), which correlate with the asymmetric formation of calcific nodules on BAV leaflets (Thubrikar et al., 1986; Sabet et al., 1999) and the asymmetric dilation patterns in BAV ascending aortas (AAs) (Fazel et al., 2008; Schaefer et al., 2008), has generated renewed support for the involvement of hemodynamic stresses in BAV disease and for the investigation of the flow in BAV aortas.

Phase contrast magnetic resonance imaging (PC-MRI) and echocardiography have revealed the existence of stress overloads in BAV aortic wall regions prone to dilation (van Ooij et al., 2015), their association with extracellular matrix dysfunction (Girdauskas et al., 2014) and their dependence on the BAV morphotype (Bissell et al., 2013). While those studies have been instrumental in providing evidence for a hemodynamic root of BAV disease, the reliability of those *in vivo* flow characterizations is challenged by the inherent lack of spatial resolution of the imaging technique and the possible hemodynamic impact of pre-existing anatomical abnormalities (e.g., dilated aorta, stenotic valve). Computational models have been designed to circumvent those limitations. Spatially resolved fluid-structure interaction simulations in intact valve-aorta geometries have demonstrated the existence of contrasted abnormalities in fluid shear stress directionality and magnitude on type-I BAV leaflets (Chandra et al., 2012), the existence of stress overloads in BAV AAs (Gilmanov and Sotiropoulos, 2016) and their ability to mediate aortic wall degeneration (Atkins et al., 2014), and the influence of the BAV cusp fusion on aortic flow abnormalities (Cao and Sucosky, 2015; Cao et al., 2017). However, the complexity of the native tissue mechanical characteristics and the native turbulent flow regime combined with the computationally demanding coupling of the fluid and structural problems are still hampering those models. On this basis, the *in vitro* approach, which aims at measuring the flow in realistic anatomies using high-resolution flow diagnostic techniques, poses as a legitimate alternative to the *in vivo* and *in silico* approaches. Particle image velocimetry (PIV) measurements in TAV and BAV tissue models have reported increased energy loss, flow turbulence and unsteadiness in BAVs as well as increased wall shear stress in BAV AAs (Saikrishnan et al., 2012; Yap et al., 2012; Seaman and Sucosky, 2014; Seaman et al., 2014). Laser Doppler velocimetry measurements performed in a physiologic flow loop have revealed increased fluid shear stress frequency on BAV leaflets relative to TAV leaflets (Yap et al., 2012). Lastly, PIV experiments in simulated calcified valve models have indicated the dependence of BAV flow abnormalities on the degree of calcification (Seaman et al., 2014). Although these flow measurements have provided a reasonable compromise between accuracy and fidelity to the native configuration, they have often implemented chemically fixed valves and rigid or simplified aorta geometries, which resulted in an approximation of the native hemodynamics.

The review of the current literature on BAV hemodynamics reveals several knowledge shortcomings, which can be articulated by the following questions: (1) *What is the initial impact of the BAV anatomy on the large-scale flow structures and wall shear stress in the native AA?* (2) *What is the influence of the BAV cusp fusion on those flow characteristics?* Therefore, the objective of the present study was to quantify and compare experimentally the pulsatile flow characteristics generated in the aortic root and AA by a TAV and the three type-I BAV morphotypes (i.e., LR-BAV, RN-BAV, NL-BAV) using PIV.

## Methods

### Valve models

**Figure 1:**
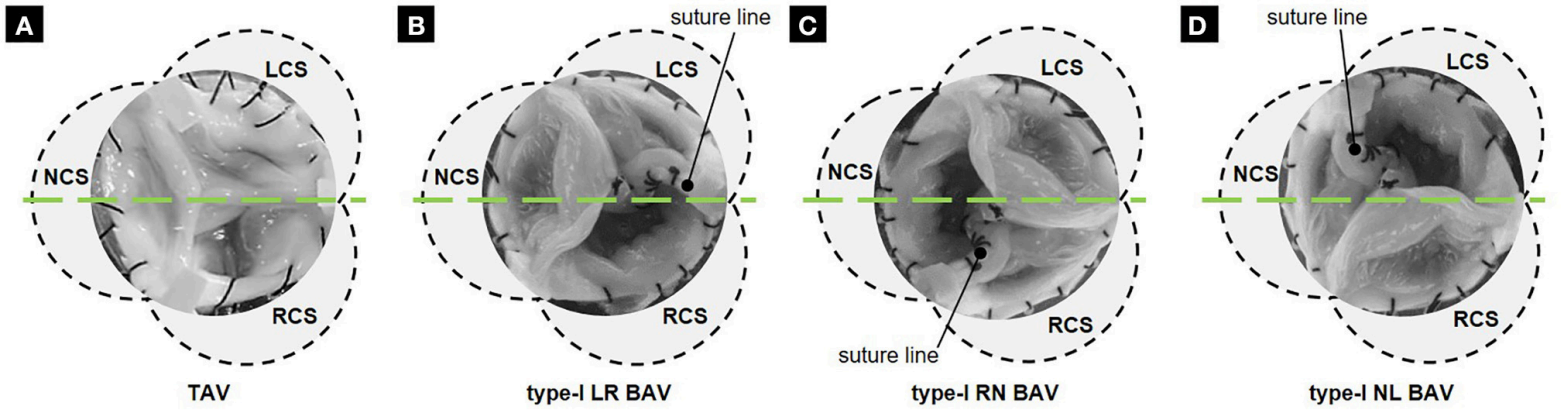
**Tissue valve models: (A)** TAV; **(B)** LR BAV; **(C)** RN BAV; and **(D)** NL BAV (RCS, right-coronary sinus; NCS, non-coronary sinus; LCS, left-coronary sinus; green line, laser sheet position 1).

Four tissue valve models were constructed to replicate a TAV anatomy and the three type-I BAV morphotypes (i.e., LR-, RN- and NL-BAV). These anatomies were selected based on their high prevalence and their common association with aortopathy (Sievers and Schmidtke, 2007). Each model was created from a normal TAV excised from a porcine heart obtained from a local abattoir. Following slaughter, the whole aortic root (i.e., aortic sinus and leaflets) was transported to the laboratory in ice-cold phosphate buffer saline (PBS). Upon arrival in the laboratory, all subsequent procedures were conducted within an hour and by frequently dipping the aortic root in PBS to keep it moist at all times. The aortic root was first trimmed to remove excess muscle and connective tissue, while preserving the narrow strip of aortic tissue along which each leaflet attaches to the wall. The resulting valve was then sutured to a circular supporting plate following our previously published protocol (Seaman et al., 2014, 2015). The BAV models were created by suturing two leaflets (left- and right-coronary leaflets for LR-BAV, non- and left-coronary leaflets for NL-BAV, and right- and non-coronary leaflets for RN-BAV) along their common free edge (Figure 1). In an effort to maintain the native mechanical properties of the leaflets, no fixative agent was used during the valve preparation or during the measurements.

### Valve and aorta chamber

**Figure 2:**
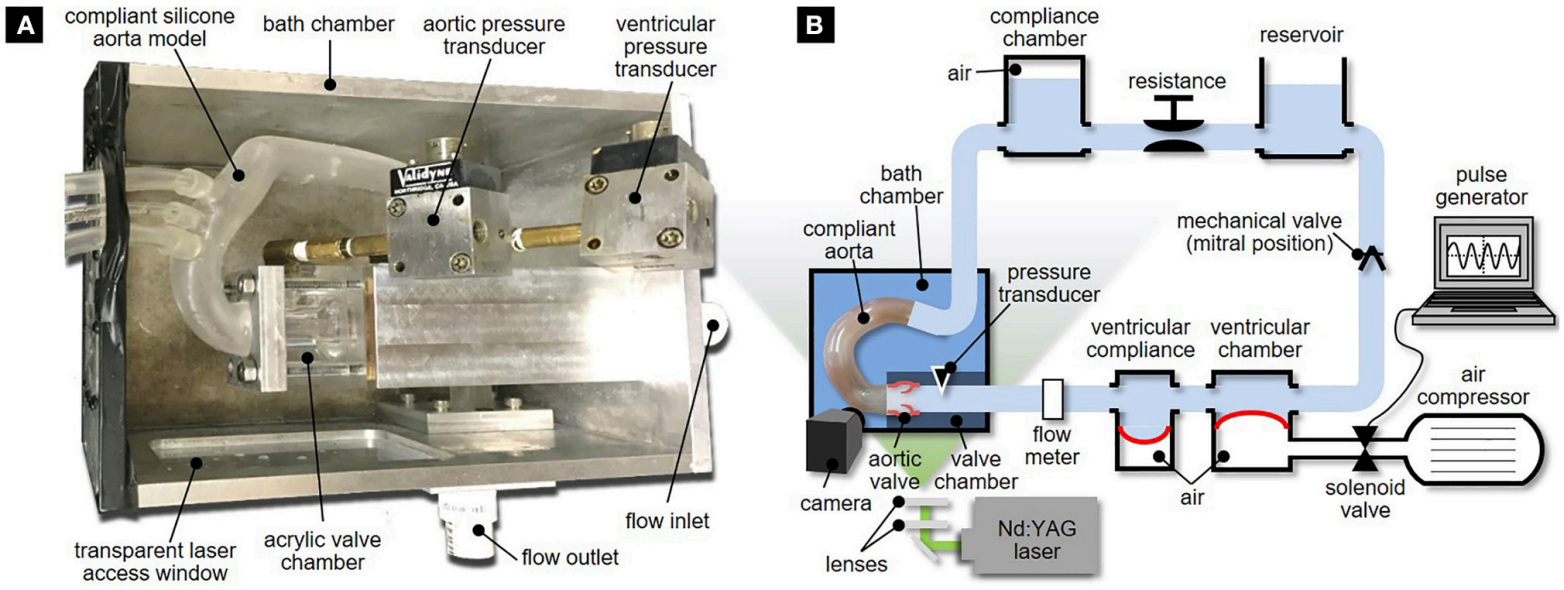
**Experimental setup: (A)** bath chamber with valve chamber and compliant aorta phantom; and **(B)** left-heart simulator.

The valve sutured on its mounting plate was placed in a valve chamber made of acrylic and constructed with flat external walls to minimize refraction of the incident laser sheet (Figure 2A). The chamber consists of an idealized three-lobed sinus geometry (Swanson and Clark, 1974; Angelini et al., 1989) and a straight cylindrical conduit (inner diameter: 24 mm; length: 20 mm) mimicking the proximal segment of the tubular AA. The valve chamber was designed to allow control over the angular position of the circular mounting plate relative to the aortic sinuses, which permitted the precise positioning of the fused leaflet for each morphotype.

The valve chamber was connected to a realistic compliant aortic arch model. The aorta geometry was reconstructed based on computed tomography images of a human aorta obtained from the Visible Human Project. This model matched the one used in our previous computational study on the effects of BAV flow on AA hemodynamics (Cao and Sucosky, 2015). The optically accessible silicone compliant model was fabricated using three-dimensional printing (Medisim Corp. Inc., Alton, ON) and featured a uniform wall thickness (2.0 ± 0.2 mm).

To enhance optical access and limit optical distortion, the aorta phantom and the valve chamber were submerged in a rectangular bath chamber filled with an index matching solution of water and glycerol (55 and 45% by volume, respectively). The properties of this mixture (density: 1060 kg/m^3^, dynamic viscosity: 3.8 cP) approximated blood properties while providing partial index matching (refractive index: 1.40) with the silicone and acrylic materials (refractive index: 1.41 and 1.49, respectively). The bath chamber features an inlet and outflow ports that connect to the inlet section of the valve chamber and the outlet section of the aorta phantom, respectively.

### Pulsatile flow loop setup

The bath chamber was mounted in a modified version of our left-heart simulator (Seaman et al., 2015). The flow loop (Figure 2B) was driven by a pulse generator consisting of an air compressor (1NNE5, Grainger, Lake Forest, IL) delivering pressurized air (35 psi) to a ventricular chamber (6NZK3 diaphragm accumulator, Parker Hannifin, Cleveland, OH) mimicking ventricular function. The filling of the ventricular chamber was controlled by a 2-position 3-way solenoid valve (56C-13-111CA, Mac Valves, Wixom, MI) whose timing was regulated by a square wave signal generated in Labview (National Instruments Corp., Austin, TX). Ventricular compliance was introduced by the inclusion of a second diaphragm accumulator (6NZK2, Parker Hannifin) just downstream of the ventricular chamber.

**Figure 3:**
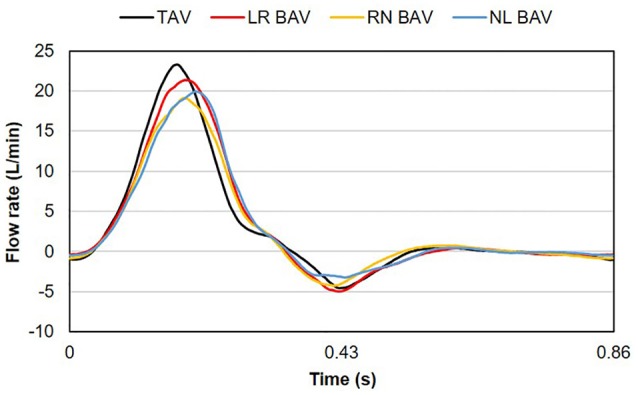
**Flow rate waveforms generated by the left-heart simulator with the four valve models**.

A fluid reservoir (volume: 4 L) fed the ventricular chamber during diastole to replicate atrial function, while enabling control over the hydrostatic pressure generated in the loop. A gate valve and a compliance chamber (volume: 1.5 L) connected downstream of the bath chamber were used to adjust vascular resistance and compliance. The instantaneous flow rate delivered by the left-heart simulator was measured downstream of the ventricular compliance chamber by an in-line ultrasonic flow meter (ME-XPN-19, Transonic, Ithaca, NY). Two pressure ports located 24 mm upstream and 24 mm downstream of the valve annulus were connected to two pressure transducers (DP15-34, Validyne Engineering Corp., Northridge, CA) to provide ventricular and aortic pressure measurements. The flow loop was tuned to generate a near physiologic aortic pressure of 135/70 mmHg at 70 beats per minutes. This condition resulted in a cardiac output of 3.1 L/min in the TAV and a smaller cardiac output between 2.8 and 3.0 L/min in the BAVs, due to their intrinsic degree of stenosis and higher resistance to the flow (Figure 3). Those levels are within the physiologic ranges reported for BAV patients (average cardiac output: 3.5 ± 1.3 L/min) (Barker et al., 2010; Mirabella et al., 2015).

### PIV setup

**Figure 4:**
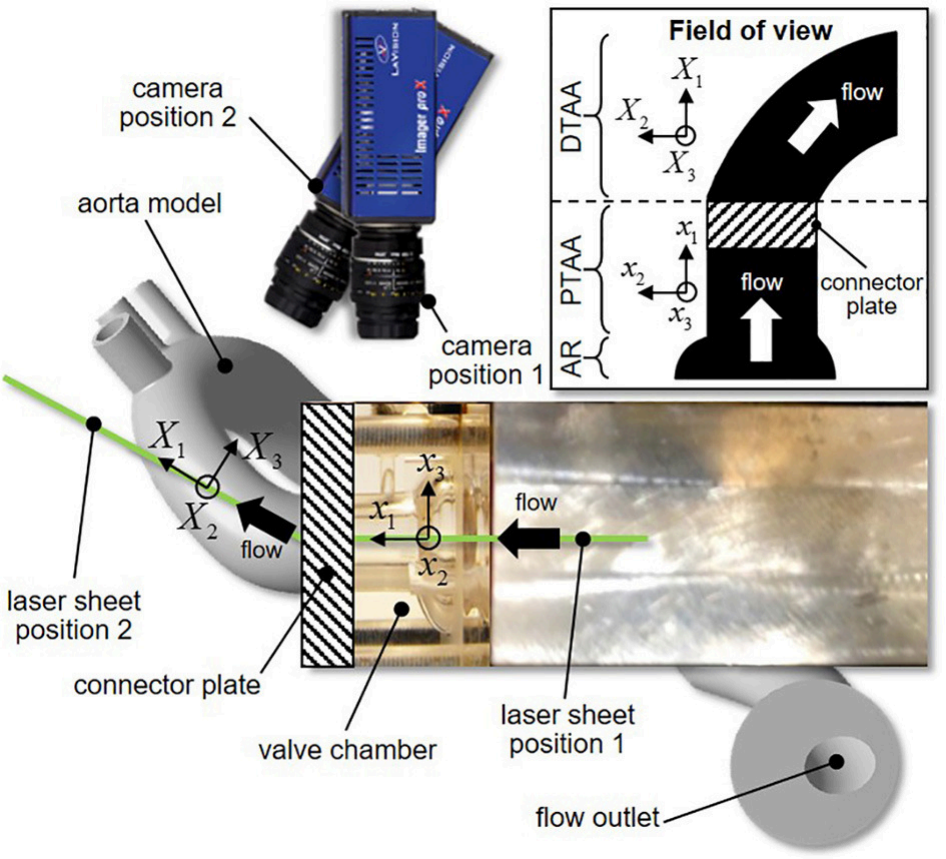
**Laser sheet and camera configurations (inset, reconstructed field of view; AR, aortic root; PTAA, proximal tubular ascending aorta; DTAA, distal tubular ascending aorta)**.

PIV was used to investigate the flow fields in the aortic root and the AA. The flow was seeded with neutrally buoyant hollow glass microspheres (Sphericel 110P8, Potters Industries LLC., Malvern, PA) with a mean diameter of 11.7 μm and a density of 1100 kg/m^3^. The PIV system (Flowmaster, LaVision, Goettingen, Germany) consisted of a double-head Nd:YAG laser (New Wave Research Solo II) generating a pulsed output beam (wavelength: 532 nm; energy: 30 mJ; pulse duration: 3–5 ns). Optical mirrors and lenses were used to form the beam into a 200 μm thick laser sheet. For each valve model, the laser sheet was positioned to illuminate two sections of the flow through a laser access window located on the side of the bath chamber. The first laser position illuminated the middle horizontal cross section of the valve chamber, while the second position illuminated the middle cross section intersecting the centerline of the silicone AA model. This setup enabled the capture of the flow characteristics in the middle cross sections of the aortic root and proximal tubular AA, as well as in the middle cross section of the distal tubular AA (Figure 4; see Figure 1 for laser position relative to each valve model). The two resulting fields of view were separated by a 10-mm long stainless steel connector plate, which blocked optical access to the flow over that region. For each laser position, a charge-coupled device camera (Imager Pro X 2M) fitted with a 60-mm lens (Micro Nikkor, Nikon Inc., Melville, NY) and narrow band pass filter (532 ± 10 nm) was placed above the bath chamber perpendicular to the laser sheet to image a 62 × 46 mm section of the flow at a resolution of 1648 × 1214 pixels. Image acquisition was performed by a 64-bit, dual channel frame grabber coupled to a dual-core, dual-processor computer. For each valve model and each laser position, image sets were collected at 20 phases of the cardiac cycle. At each phase, 415 image pairs were captured by the camera in phase-locked mode. Briefly, for the first phase, image acquisition and laser pulsing were synchronized with the opening of the solenoid valve regulating the filling of the ventricular chamber. For all other phases, a delay was imposed to capture the image pairs at the desired phase of the cardiac cycle. The image pairs were cross-correlated in Davis 7.2 (LaVision) using a multi-pass scheme with an initial interrogation window of 64 × 64 pixels with a 50% overlap and a final interrogation window of 8 × 8 pixels with a 50% overlap, which permitted to achieve a spatial resolution of 300 μm.

### Hemodynamic characterization

The in-plane instantaneous velocity fields **u**(**x**, *t*) obtained by cross-correlation were first filtered to eliminate erroneous velocity vectors and then ensemble-averaged over 415 realizations to yield an average velocity field **u**(**x**, *t*) at each phase. All subsequent analyses were performed in Tecplot 360 (Tecplot Inc., Bellevue, WA). The velocity fluctuations **u**′(**x**, *t*) were obtained by Reynolds decomposition:

(1) $$u\prime (x,t)=u(x,t)-\bar{u}(x,t).$$

**Figure 5:**
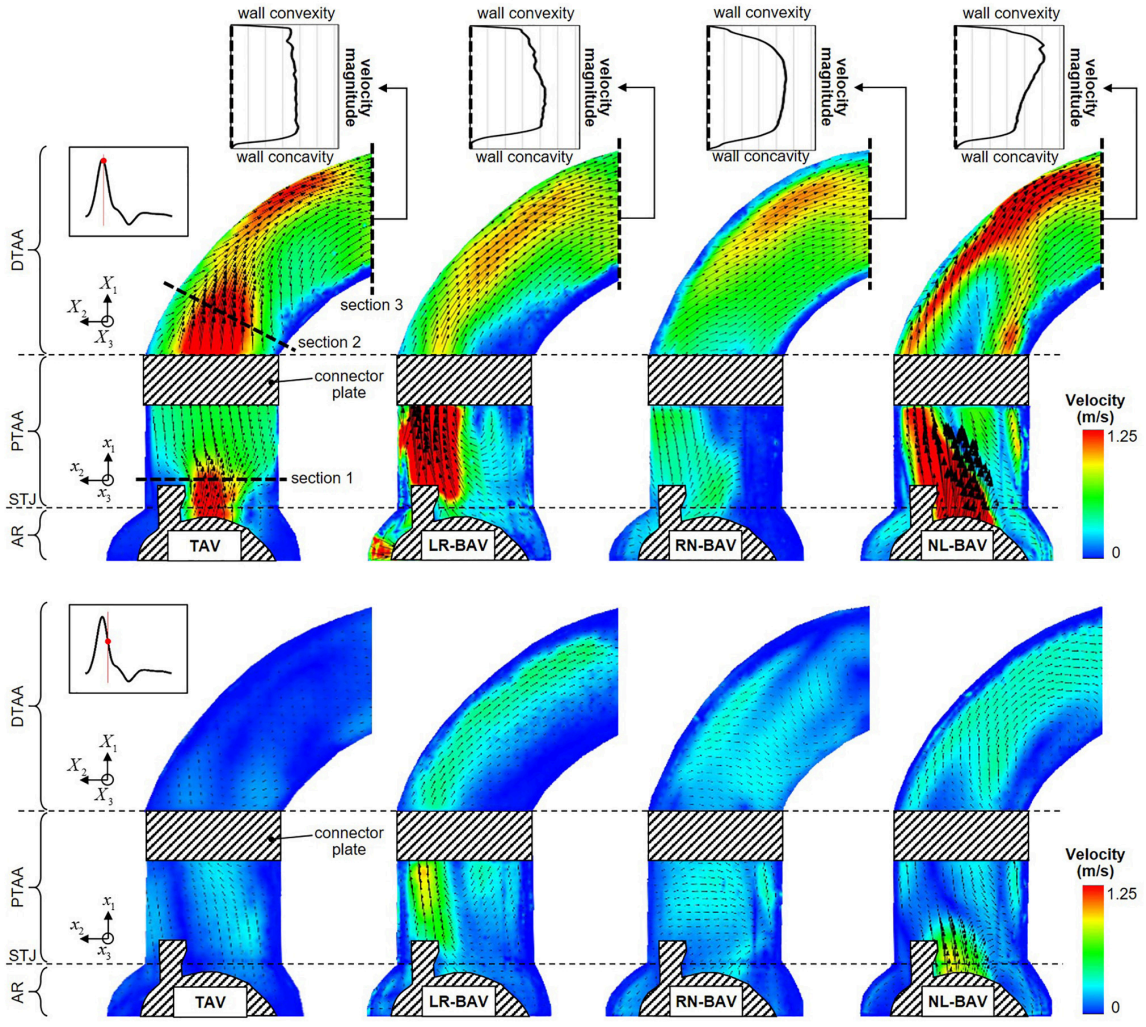
**Snapshots of the mean velocity fields generated by the four valve models at peak systole (top) and late systole (bottom), and peak-systolic velocity profiles measured across the outlet section of the aorta phantom (AR, aortic root; STJ, sinotubular junction; PTAA, proximal tubular ascending aorta; DTAA, distal tubular ascending aorta)**.

The viscous shear stress τ(**x**, *t*) was calculated in Tecplot as

(2) $$\bar{\tau }(x,t)=\mu \left(\frac{\partial {\bar{u}}_{1}(x,t)}{\partial x_{2}}+\frac{\partial {\bar{u}}_{2}(x,t)}{\partial x_{1}}\right),$$

where μ is the fluid dynamic viscosity. Turbulence characteristics were quantified in terms of the Reynolds shear stress τ′(**x**, *t*) defined as

(3) $$\tau \prime =\rho \bar{u\prime_{1}(x,t)u\prime_{2}(x,t)},$$

where ρ is the fluid density. In addition, consistent with previous flow analyses on patient MRI data, flow skewness and eccentricity were measured in three sections located 4 mm downstream of the sinotubular junction (section 1 in Figure 5), in the middle AA (section 2 in Figure 5), and in the distal section of the tubular AA (section 3 in Figure 5). The skewness of the systolic valvular jet was assessed in terms of the valve flow angle (θ) defined as

(4) $$\theta =\cos^{-1}\left(n\cdot Q\right),$$

where **Q** is the mean flow vector and **n** is the unit vector normal to the aortic section of interest (Mahadevia et al., 2014). The eccentricity of the systolic valvular jet was characterized in terms of the flow displacement (*d*), i.e., the distance between the center of the aortic section of interest and the centroid of the top 15% of velocities in the same section (Sigovan et al., 2011). These two metrics were calculated on the peak-systolic flow field.

## Results

### Jet skewness, eccentricity and mean velocity field

The mean velocity fields **u**(**x**, *t*) measured at peak systole and early diastole for all valve models are shown in Figure 5. Peak-systolic flow angle and flow displacement values for each valve are reported in Table 1. At peak-systole, the TAV generates an orifice jet essentially aligned along the axis of the aorta. Further downstream, the flow patterns and velocity vectors follow the curvature of the tubular AA smoothly. These observations are supported by the moderate flow angle and flow displacement (6.3 < θ < 14.1°, 0.6 < *d* < 1.4 mm) measured throughout the geometry. In contrast, the BAVs generate orifice jets skewed toward the convexity of the tubular AA. While this phenomenon is obvious for the LR-BAV, it is less pronounced in the RN- and NL-BAV cases, due to the orientation of the RN- and NL-BAV jets out of the plane of observation. The apparent skewness of the NL-BAV jet toward the wall convexity is a technical artifact due to the inability of the two-dimensional PIV plane to capture the full three-dimensional helical flow characteristics generated by this morphotype in the aorta. The combination of the larger flow angles and displacements generated by the BAVs (3.2 < θ < 37.1°, 0.3 < *d* < 8.2 mm) forces the jet to impinge the AA wall at locations proximal to the TAV jet impingement site. In addition, the initial flow asymmetry observed in the three BAVs generates a recirculation zone near the concavity of the proximal tubular AA, whose direction depends on the morphotype (LR-BAV and RN-BAV: clockwise; TAV and NL-BAV: counterclockwise).

**Table 1:** **Peak-systolic flow angle and displacement**.

	**Measurement site**	**TAV**	**LR-BAV**	**RN-BAV**	**NL-BAV**
θ (°)	Section 1	14.1	18.4	23.4	3.2
	Section 2	6.3	15.6	37.1	6.3
	Section 3	13.7	12.4	14.8	12.7
*d* (mm)	Section 1	1.4	6.8	1.1	4.0
	Section 2	0.6	6.0	7.2	8.2
	Section 3	1.2	−0.4	−0.3	5.8

While the three morphotypes generate a jet-like flow structure characterized by a high-velocity core and a low-velocity mixing zone near the valve orifice, some interesting variations can be observed downstream. In the RN-BAV and NL-BAV, the skewed orifice jet formed in the aortic root separates into two branches near the inlet of the distal tubular AA before remerging near the outlet, suggesting the existence of recirculation and out-of-plane motion. In contrast, the LR-BAV flow maintains a jet-like structure up to the distal section of the tubular AA. The progressive development of the flow along the axis of the aorta tends to normalize the flow and eliminate the differences observed upstream as suggested by the nearly similar velocity profiles, low flow angles (θ < 1.8°, *d* < 5.8 mm) and displacements measured at the outlet section. Lastly, the analysis of the in-plane peak-systolic velocity magnitude also reveals important differences between the valves. While the TAV maintains a normal peak-systolic velocity (1.7 m/s), the LR-BAV and NL-BAV peak velocities (2.9 and 3.9 m/s, respectively) fall in the stenotic range.

### Viscous shear stress

**Figure 6:**
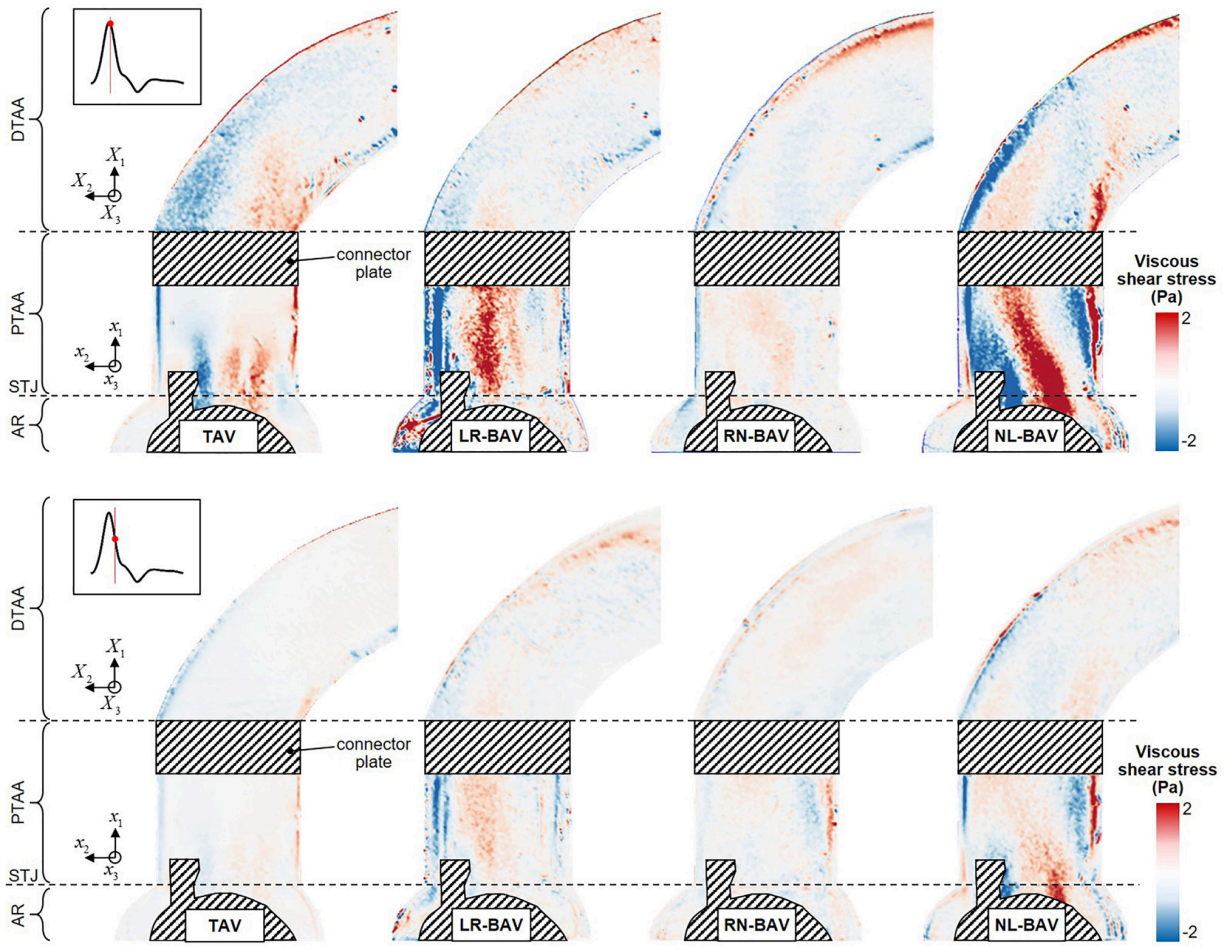
**Snapshots of the viscous shear stress fields generated by the four valve models at peak systole (top) and late systole (bottom) (AR, aortic root; STJ, sinotubular junction; PTAA, proximal tubular ascending aorta; DTAA, distal tubular ascending aorta)**.

The peak-systolic and early diastolic viscous shear stress fields τ(**x**, *t*) are shown in Figure 6. At peak systole, the shear stresses are concentrated in the shear layers that extend from the tip of the leaflets. The skewness of the LR- and NL-BAV jets toward the convexity of the proximal tubular AA subjects the aortic wall to a 1.7-fold increase in shear stress magnitude relative to the TAV. However, while the region of wall shear stress overload remains constrained in the proximal tubular AA in the LR-BAV case, it localizes in the distal tubular AA in the NL-BAV case. During the deceleration phase, the convexity of the TAV proximal aorta experiences a substantial reduction in wall shear stress (72% reduction relative to peak-systole). This is not the case with the LR- and NL-BAV aortas, in which the complex rotational flow structures subject the wall to sustained wall shear stress overloads (2.0-fold increase relative to the TAV) despite the reduction in forward flow momentum. The RN-BAV, which generates an orifice jet outside the measurement plane, subjects the convexity of the aortic wall to milder viscous shear stresses in the plane of observation.

### Reynolds shear stress

**Figure 7:**
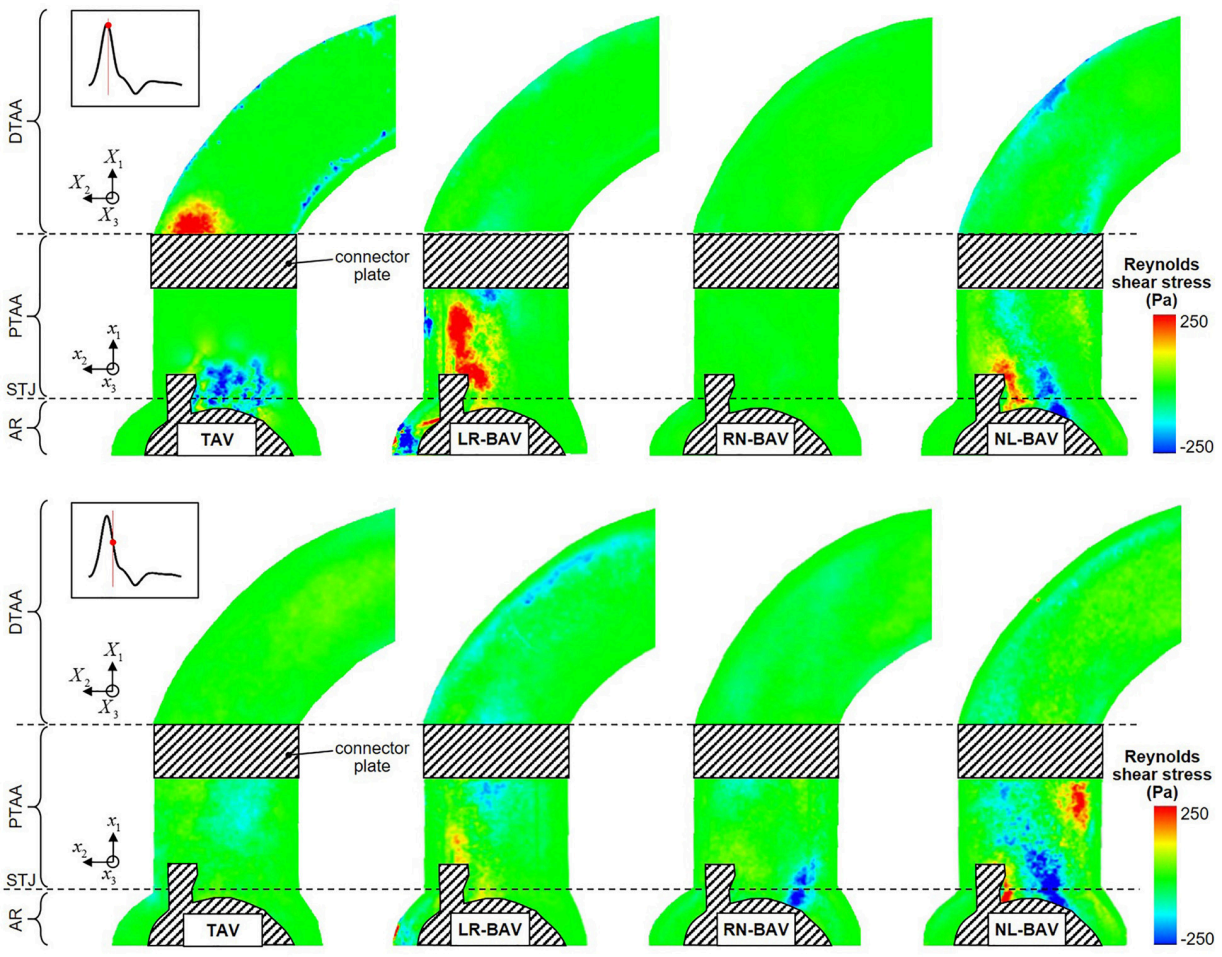
**Snapshots of the Reynolds shear stress fields generated by the four valve models at peak systole (top) and late systole (bottom) (AR, aortic root; STJ, sinotubular junction; PTAA, proximal tubular ascending aorta; DTAA, distal tubular ascending aorta)**.

The peak-systolic and early diastolic Reynolds shear stress fields τ′(**x**, *t*) are shown in Figure 7. Regardless of the valve anatomy, the peak Reynolds shear stress is two-orders-of-magnitude larger than the peak viscous shear stress, indicating the domination of the flow by the turbulent stresses. As expected, those effects are the most apparent in the wake of the leaflets, where turbulence effects and velocity fluctuations attain their maximum. The only exception to this observation is for the RN-BAV due to the orientation of the jet out of the plane of observation. Turbulent stress levels are substantially more moderate in the distal section of the AA, which suggests the possible relaminarization of the flow in this region. Consistent with the viscous shear stress measurements, the comparison of the Reynolds shear stress fields at peak systole and during deceleration reveals that turbulence dominates the flow only at peak systole in the TAV aorta (49% reduction in maximum Reynolds shear stress between peak systole and deceleration), while it is sustained over a longer period in the LR-BAV and NL-BAV aortas (24% difference in maximum Reynolds shear stress between peak systole and deceleration).

## Discussion

This *in vitro* study implemented PIV to characterize morphotype-dependent flow abnormalities in BAV aortas, prior to dilation. The results complement previous demonstrations of the existence of flow abnormalities in BAV aortas by revealing: (1) the existence of different degrees of flow abnormalities in dilated and non-dilated BAV aortas, and (2) the existence of viscous shear stress overloads in non-dilated BAV aorta regions prone to aortopathy.

### Summary of morphotype-dependent flow abnormalities

The use of the same aorta geometry and flow conditions in all our experiments permits to isolate the critical impact of the BAV morphotype on hemodynamics. First, at least two BAV morphotypes (LR- and NL-BAV) generated some degree of hemodynamic stenosis as quantified by jet velocity. While the RN-BAV jet velocity was in the normal range, measurements of the three velocity components in multiple planes would be needed to determine whether this morphotype was normo-functional or stenotic like the two other morphotypes. Nevertheless, the existence of intrinsic stenosis in BAVs is consistent with clinical reports that have estimated that nearly 50% of all BAVs exhibit some level of stenosis, without the presence of calcification (Keane et al., 2000; Sievers and Schmidtke, 2007; Hope et al., 2010). Second, the type of leaflet fusion was shown to affect primarily the site of impingement of the valve orifice jet on the aortic wall, with the LR-BAV jet impinging on the proximal convexity and the NL-BAV jet impinging further downstream in the distal proximity of the aortic wall. This morphotype-dependence resulted in different sites of viscous shear stress overload. Third, the different BAV morphotypes affected both flow eccentricity and skewness to different extents and in different regions of the AA. The results suggest that the RN-BAV generated the most pronounced abnormality in flow angle throughout the AA, although this conclusion should be considered carefully since RN-BAV flow was mostly out of the plane of observation in the experiments. In contrast, the LR- and NL-BAV morphotypes had a greater impact on flow eccentricity, with the LR-BAV generating abnormalities in the proximal and middle AA and the NL-BAV in the middle and distal AA. Lastly, the type of leaflet fusion also affected the extent and spread of flow abnormalities in the AA. In fact, while LR-BAV flow abnormalities were mostly contained within the proximal AA and progressively attenuated as the flow developed in the distal AA (16% and 12% reduction in flow angle and displacement, respectively), RN- and NL-BAV flow abnormalities amplified as the flow developed from the proximal to the distal section (up to 97 and 555% increase in flow angle and displacement, respectively). These morphotype-dependent flow features, which essentially coincide with the morphotype-dependent expression of aortopathy, may play a role in BAV aortopathy initiation and development.

### Implications for BAV aortopathy

The viscous shear stress levels measured in this study are in agreement with those from previous experimental and computational studies (Weston et al., 1999; Barker et al., 2012; Chandra et al., 2012; Meierhofer et al., 2013; Seaman et al., 2014, 2015) and have been suggested as a possible driver of aortopathy (Barker et al., 2012; Atkins and Sucosky, 2014; Atkins et al., 2014, 2016b). The present experimental results confirm the existence of shear stress overloads in aortic wall regions prone to dilation, even in normal non-dilated aortas. While this observation only suggests the potential involvement of hemodynamics in the pathogenesis of BAV aortopathy, it provides a more solid evidence of the existence of a hemodynamic pathway of BAV aortopathy when the results are put in the perspective of previous *ex vivo* and clinical studies. In fact, it is well known that 1) BAVs are associated with morphotype-dependent dilation patterns (Fazel et al., 2008; Schaefer et al., 2008); and 2) BAVs generate morphotype-dependent flow abnormalities in regions prone to dilation (as shown in the present study and in Hope et al., 2014; Cao and Sucosky, 2015; van Ooij et al., 2015; Fedak et al., 2016; Cao et al., 2017). The possible causality between these two facts has been partially but rigorously provided by *ex vivo* studies conducted in our laboratory, which have demonstrated the ability of the stress abnormalities generated in the disease-prone convexity of the LR-BAV aorta to trigger aortic medial remodeling via MMP-dependent pathways and the absence of any significant remodeling in response to the hemodynamics of the disease-protected concavity (Atkins and Sucosky, 2014; Atkins et al., 2014, 2016b; Sucosky, 2014). In this context, the data presented in this study suggests that the elucidation of the shear stress environment in BAV AAs might be critical toward the development of improved clinical guidelines for the management of BAV patients (Atkins et al., 2016a).

### Impact of aortic dilation on BAV hemodynamics

An important novelty of the present study is its particular focus on the impact of BAV flow in non-dilated aortas. The rationale for this investigation is supported by the need to determine whether the flow abnormalities typically present in BAV aortas are the consequence of the abnormal valve anatomy or a dilated aorta. Similarly to previous *in vivo* results, which may have included dilated BAV aortas (Bissell et al., 2013; Mahadevia et al., 2014), the present experimental study confirms the skewness of the BAV orifice jet toward the convexity of the aortic wall, the dependence of the degree of jet skewness on the BAV morphotype and the existence of viscous shear stress overloads in the convexity of BAV aortas. Interestingly, the absence of dilation in the present study did not systematically attenuate the degree of hemodynamic abnormality captured *in vivo* in dilated BAV aortas. This is particularly apparent for the in-plane flow skewness, which was 38% smaller in the non-dilated LR-BAV aorta but 69% larger in the non-dilated RN-BAV aorta as compared to their dilated counterparts (Mahadevia et al., 2014). Lastly, the present experimental results confirm the existence of shear stress overloads in BAV aortic wall regions prone to dilation, but also demonstrate that those abnormalities exist prior to dilation. These observations are supported by previous computational results suggesting the possible impact of aortic dilation on aortic flow (Cao et al., 2017). Therefore, while the present results confirm the morphotype-dependence of flow abnormalities in BAV aortas, they also suggest the alteration of this dependence throughout the course of the disease and the synergistic effects of BAV anatomy and aorta anatomy on aortic flow.

### Limitations

Although the experiments were carried out with the upmost rigor, the experimental technique and methodology include a few limitations. First, PIV only permitted to capture the flow in a two-dimensional section and was not able to provide more insights into the three-dimensional flow structures. While this is a known limitation of the PIV technique, it did not prevent the demonstration of flow differences between the different valve models investigated. However, this limitation combined with the relatively poor performance of PIV in quantifiying near-wall flow regions may explain some of the differences in flow and viscous shear stress between the present study in a normal aorta and previous *in vivo* studies in potentially dilated BAV aortas (Piatti et al., 2017). Second, the pressure conditions generated within the flow loop only approximated physiologic levels due to the partial replication of the native systemic compliance and resistance. However, the resulting cardiac outputs remained within the physiologic range. More importantly, all valve models were tested under the same pressure conditions in order to allow for the direct comparison of the flow results and the effective isolation of the impact of the valve morphotype. Third, the flow characterization was based on one specimen for each valve anatomy. Therefore, the flow results reported in the present study may not be fully representative of the hemodynamic abnormalities generated by each valve type. While a larger sample size would enable the production of statistically meaningful data, the use of a single specimen per valve was motivated by the requirement to obtain spatially and temporally resolved flow measurements while limiting processing time (192 h for each valve) and data storage requirements (1.5 TB for all raw images and processed velocity fields). Lastly, although the benchtop flow loop was able to generate near-physiologic pulsatile flow conditions, it included some geometrical and functional idealization (e.g., non-compliant three-lobed aortic sinus, uniaxial left-ventricular contraction) that did not replicate exactly the native anatomical characteristics of the aortic sinus and the native deformation of the left ventricle. However, the use of approximated but similar left ventricular outflow condition and sinus geometry in all experiments permitted to achieve our central objective to isolate the impact of valvular anatomy on aorta hemodynamics.

## Conclusion

This experimental study isolated for the first time the impact of the BAV morphotype on aortic flow in a compliant and realistic aorta geometry. The results demonstrate the impact of leaflet fusion on downstream hemodynamics and reveal substantial differences with respect to *in vivo* studies on dilated aortas. Most significantly, all BAV morphotypes subject the aortic wall to shear stress overloads at locations prone to dilation, providing more support for the existence of a hemodynamic etiology in BAV aortopathy.

## Author contributions

AMN and AM performed the experiments, analyzed the data and wrote the paper. PS analyzed the data, wrote the paper and conceived the work.

## Funding

Research described in this paper has been funded by the National Science Foundation (CAREER CMMI-1148558), the American Heart Association (17GRNT3350028, 11SDG7600103, and 14PRE18940010).

### Conflict of interest statement

The authors declare that the research was conducted in the absence of any commercial or financial relationships that could be construed as a potential conflict of interest.
